# Photon counting detector CT-derived virtual non-contrast images of the liver: comparison of conventional and liver-specific algorithms across arterial and portal venous phase scans

**DOI:** 10.1186/s12880-025-01849-0

**Published:** 2025-08-04

**Authors:** Anna-Katharina Gerstner, Franka Risch, Luca Canalini, Gerlig Widmann, Elke R. Gizewski, Stefanie Bette, Simon Hellbrueck, Thomas Kroencke, Josua A. Decker

**Affiliations:** 1 Department of Radiology, Medical University Innsbruck, Anichstrasse 35, Innsbruck, 6020 Austria; 2 Department of Diagnostic and Interventional Radiology, University Hospital Augsburg, Stenglinstr. 2, 86156 Augsburg, Germany

**Keywords:** Virtual non-contrast images, VNC, TNC, Photon counting CT, “Liver VNC”, “Virtual unenhanced”, BMI

## Abstract

**Background:**

The aim of this retrospective study is to compare photon-counting detector computed tomography (PCD-CT) derived virtual non-contrast (VNC) images of the liver reconstructed from both arterial and portal venous phase using conventional and liver-specific VNC algorithm to true non-contrast images, in context of the body mass index (BMI).

**Methods:**

VNC images reconstructed from multiphase (non-contrast, arterial and portal venous phase) PCD-CT scans performed between April 2021 and February 2023 were analysed retrospectively. For each patient, four VNC series were generated: two series (arterial and portal venous) using a conventional VNC algorithm (VNC_conv_^art^; VNC_conv_^pv^) and two using a liver-specific “Liver VNC” algorithm (VNC_Liver_^art^; VNC_Liver_^pv^). Regions of interest were placed in the left and right liver lobes and in the spleen, avoiding large vessels and focal lesions. The VNC CT-values were then compared to those of the corresponding true non-contrast images (TNC). The subsequent analysis involved the calculation of both correlation and mean offsets. The median split was utilised to ascertain distinct cohorts of patients with elevated and reduced body mass indices. These cohorts were then subjected to a comparative analysis of attenuation values to discern potential disparities between them. The results were compared by using parametric and non-parametric tests; Pearson’s correlation coefficient was employed. Bland-Altman plots were utilised to visually assess the agreement between results and Passing-Bablok regression, thereby quantifying the observed agreement.

**Results:**

The study population comprised 42 patients (mean age 70.0 ± 10.2 years, 33 males). Mean offsets between TNC and VNC_conv_^art^ was 0.62 ± 5.23 HU, TNC-VNC_conv_^pv^ 1.24 ± 6.67 HU, TNC-VNC_Liver_^art^ -0.94 ± 5.59 and TNC-VNC_Liver_^pv^ -0.35 ± 6.99 with no significant difference. Significant differences were found for VNC_conv_^art^, VNC_conv_^pv^ and VNC_Liver_^art^ images regarding spleen attenuation. Bland-Altman plots demonstrated good agreement and the absence of any systematic difference in liver attenuation. As for the TNC-VNC_conv_^art^, TNC-VNC_conv_^pv^, TNC-VNC_Liver_^art^ and TNC-VNC_Liver_^pv^ variables, strong correlations were obtained (Pearson’s coefficient: 0.79, 0.69, 0.79 and 0.7, all *p* < 0.001). The investigation revealed no statistically significant disparities between the BMI groups with respect to the mean offset of liver density (*p-value*:TNC-VNC_conv_^art^ 0.51; VNC_conv_^pv^ 0.61; VNC_Liver_^art^ 0.68; VNC_Liver_^pv^ 0.45). Furthermore, no significant offset between TNC and VNC images was detected within each BMI group. A Passing-Bablok regression analysis revealed no systematic or proportional difference between the two methods.

**Conclusion:**

It is evident that PCD-CT-derived VNC images generally constitute a corresponding alternative to TNC images. However, caution is advised in the interpretation of images, as there are outliers with differences exceeding 15 HU are present. In general, the mean values obtained from the analysis of, VNC images reconstructed from arterial and portal venous phases employing both the liver-specific and general VNC reconstruction algorithm did not demonstrate any clincially significant difference when compared with TNC images. Furthermore, no significant discrepancy was observed in the utilisation of the conventional and the liver-specific algorithm. The findings of this study demonstrated that, within the limitations of the study, the patients’ BMI did not have a significant impact on the VNC images.

**Supplementary Information:**

The online version contains supplementary material available at 10.1186/s12880-025-01849-0.

## Background

With the implementation of photon-counting detector computed tomography (PCD-CT) and the associated post-processing algorithms, CT imaging has undergone a significant advancement. PCD-CT provides spectral data for a range of post-processing techniques [1]. The ability to detect and weigh individual photons according to their energies, it enables spectral separation and more accurate multi-material decomposition in comparison to energy-integrating CT [1–5]. The application of energy-thresholding has been demonstrated to be an effective method of suppressing electronic noise [2, 6]. This suppression can be instrumental in enhancing the quality of post-processing reconstructions, such as VNC. It is evident that PCD-CT inherently acquires spectral data. Consequently, VNC images can be reconstructed retrospectively without the necessity for an a priori determination of a dual energy protocol. Two VNC post-processing algorithms are commercially available for soft-tissue applications: a specific liver VNC and a general VNC algorithm. Both use voxel-based material separation. The liver VNC algorithm operates under the assumption of every voxel contains only fat, soft tissue and iodine [7].

One of the most regularly used post-processing algorithms is the VNC algorithm [8, 9]. The existing offers equivocal results with regard to the question of consistency of attenuation values on VNC images. Some studies only show slight systematic deviations between VNC and TNC with good agreement between TNC and VNC [10–12]. As stated in other studies, clinically relevant systematic differences of more than 10 Hounsfield Units (HU) have been observed [9]. VNC images reconstructed from portal venous phase have been reported to be superior compared to arterial phase VNC series in cases of active bleeding [13]. Furthermore, these offsets can be observed with the liver-specific algorithm demonstrating a slight advantage [14]. It is important to ascertain the consistency of the ‘LiverVNC’ algorithm in comparison and conventional algorithms in comparison to true non-contrast images (TNC) given the evident disparities.

It is widely acknowledged that the BMI has an effect on the acquisition of CT images. It is imperative that acquisition parameters are adapted in order to achieve images of diagnostic value, particularly in cases involving patients with a BMI > 30 kg/m^2^. As demonstrated by *Durieux et al.*, a significant offset was identified between VNC and TNC for a third-generation Dual Energy CT (DECT). These differences were significantly and positively correlated with the BMI [7].

As previously outlined, VNC algorithms yield valuable images [10, 11, 15]. Nevertheless, ambiguities persist concerning the scanning phase and post-processing algorithm. The objective of this study is to assess the impact of phase and reconstruction algorithms, as well as BMI, on PCD-CT-VNC images of the liver. These images are reconstructed from arterial and portal venous phases and generated by a conventional and a liver-specific post-processing algorithm. It is hypothesised that VNC-derived attenuation values do not deviate significantly from true non-contrast values across a clinical cohort.

## Methods

### Patient selection

This retrospective study was carried out at the University Hospital Augsburg, Bavaria, Germany. It was approved by the local institutional review board (Ludwig Maximilian University LMU Munich, project no 22–0456) and informed consent was waived due to the retrospective study design. All scans were performed for diagnostic use in accordance with clinical standard protocols. Patients’ characteristics were obtained from the electronic medical records and patients´ data were anonymised.

**Fig. 1:**
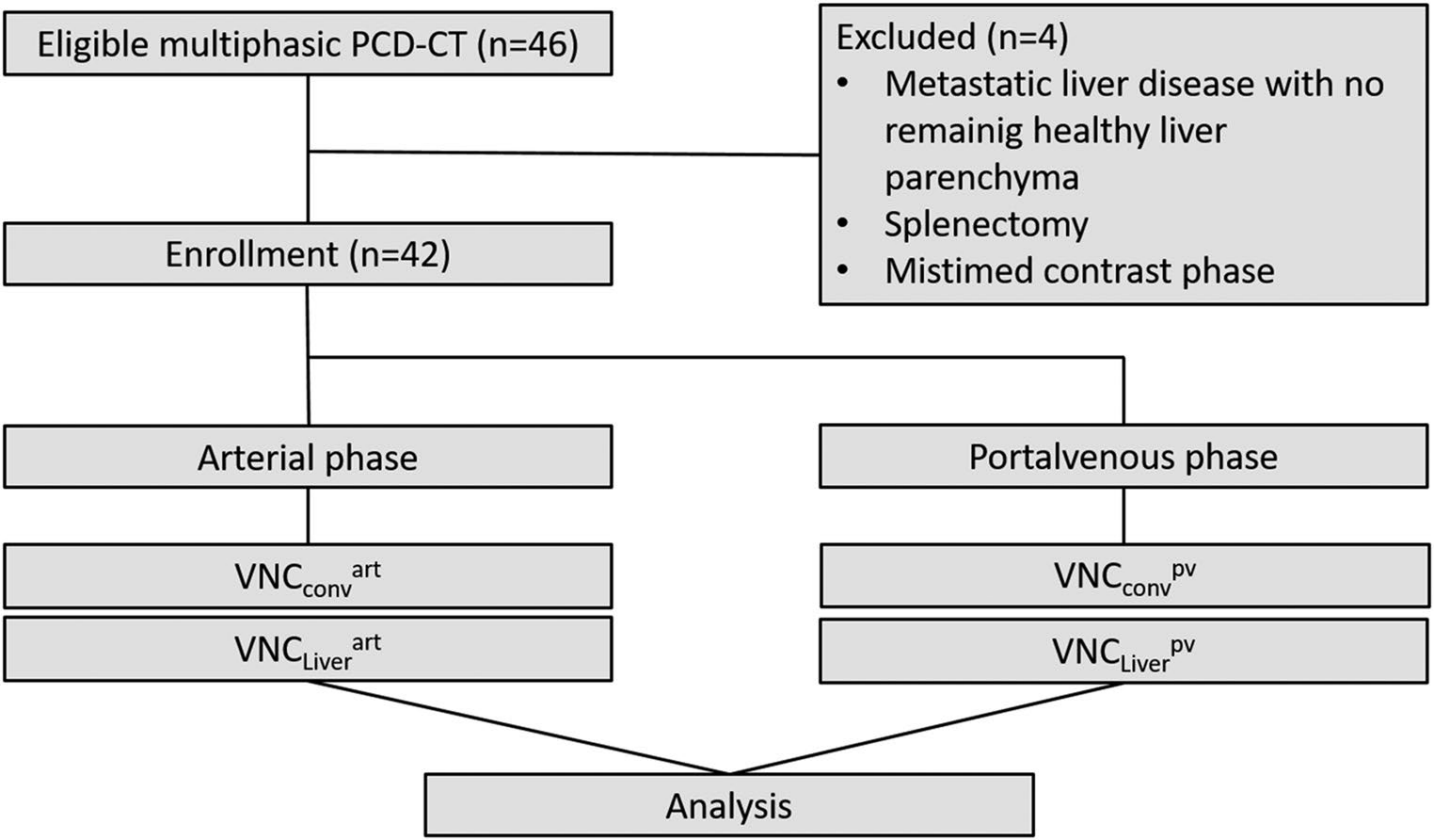
Flowchart of patients’ selection process. Note: 42 of previously selected 46 patients were enrolled. For every enrolled patient 4 VNC images were available, reconstructed by “LiverVNC” and “Virtual Unenhanced” algorithm separately for arterial and portal venous phase

A comprehensive review of all PCD-CT scans was conducted over the period spanning from April 2021 and February 2023. In order to reflect the general applicability of VNC in clinical routine, the minimal requirement for inclusion was the availability of a non-contrast scan followed by an arterial, and a portal venous phase. No preliminary selection was made with regard to the underlying liver disease. A total of 1761 examinations were identified. The majority of cases were excluded on the grounds of inadequate scanning areas (liver not fully included) and inadequate scanning protocols (e.g. only portal venous phase or non-contrast scan available, missing true non-contrast scan) or repetitive scans of the same patient with remaining 46 patients. Patients with splenectomy were excluded. The patient`s selection process is illustrated in Fig. 1.

### CT protocol, image acquisition and radiation dose

All CT scans were obtained using a first-generation dual-source PCD-CT (NAEOTOM Alpha; Siemens Healthineers, Erlangen, Germany). The spectral acquisition technique was conducted with a collimation of 144 × 0.4 mm and a default setting of detector-based primary thresholding of 20, 35, 65, and 70 keV-spectral energy binning for spectral separation was used.

The eligible examinations were performed for different indications, mainly for assessing malignant tumours of the liver. Depending on the indication, the scan range covered the upper abdomen or the whole trunk. Pitch factor was 0.8 and rotation time 0.5. All patients were examined in a supine position and unenhanced scan acquisition was obtained first.

Non-contrast and contrast-enhanced scans were performed at 120 kV. Iodinated contrast medium (100 ml Iopromide, Ultravist 300, Bayer, Leverkusen, Germany) was administered via intravenous contrast injection followed by a 30 ml saline bolus both using a 4.0 ml/s flow rate. The scans were bolus-triggered within the ascending or abdominal aorta (after an attenuation of 120 HU) with a delay of 10 s for arterial contrast phase and 75 s for portal venous contrast phase. Dose Length Product (DLP) and volumetric CT Dose Index (CTDI_Vol_) were used and retrieved from the automatically archived dose reports.

All images were reconstructed in axial view with the same slice thickness (2 mm) using a quantitative kernel (Qr40) with iterative reconstruction (QIR3).

### Image reconstruction

**Fig. 2:**
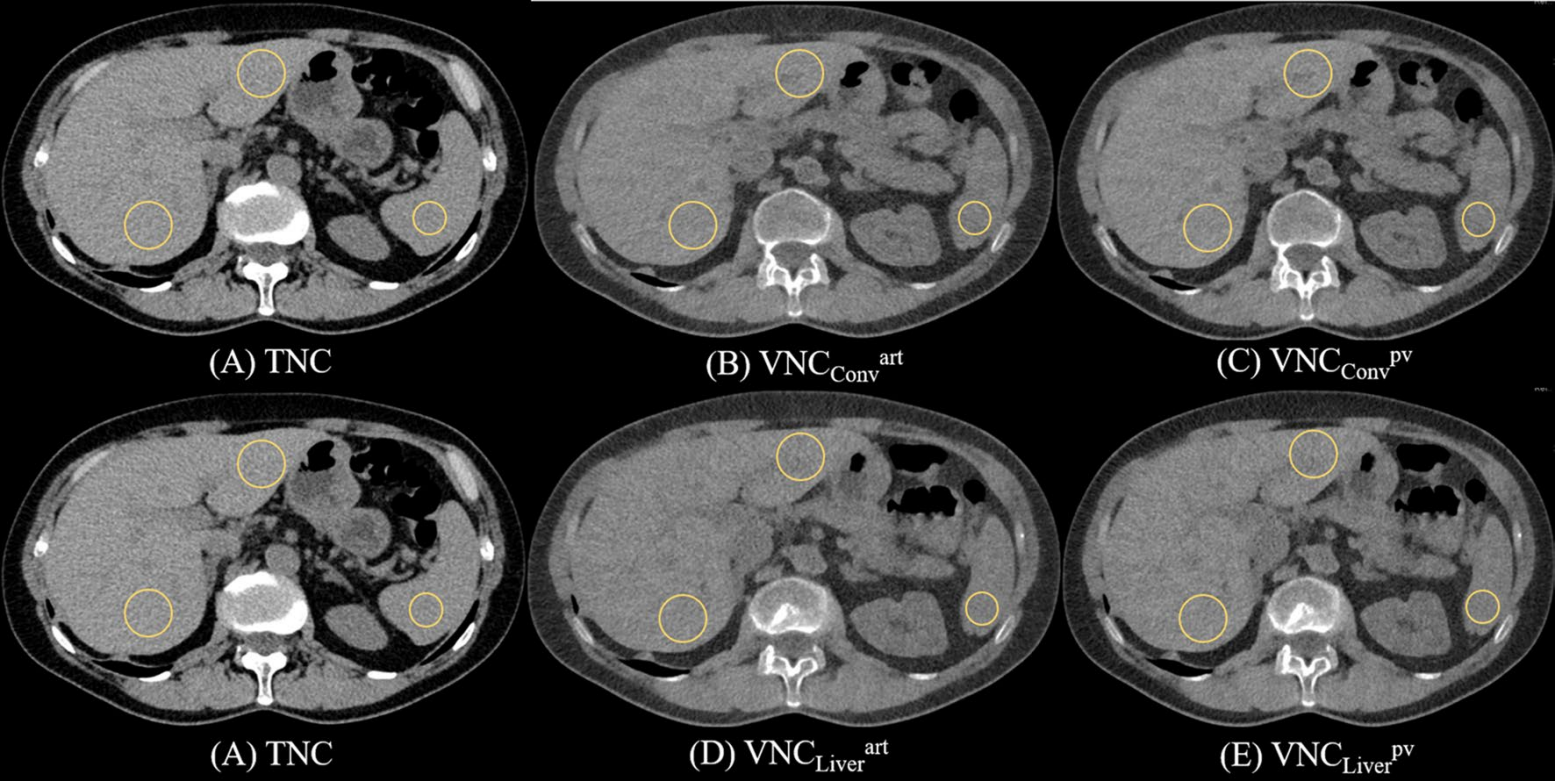
TNC and VNC Images. Note: True non-contrast CT scan (**A**) with corresponding virtual non-contrast images derived from arterial phase and reconstructed with VNC_conv_ (**B**) and VNC_Liver_ algorithm (**C**). Images from portal venous phase and reconstructed with VNC_conv_ (**D**) and VNC_Liver_ algorithm (**E**)

A dedicated working station (DualEnergy application, Syngo.Via, VB60 version, Siemens Healthineers, Erlangen, Germany) was used to retrospectively analyse the spectral datasets. VNC series were reconstructed from the arterial phase as well as from the portal venous phase using the software applications “LiverVNC”, a dedicated algorithm adapted for the liver [14] as well as the conventional “Virtual Unenhanced” VNC algorithm. Both algorithms use three material decomposition techniques with each voxel containing up to three different materials. The “LiverVNC” algorithm works on the supposition of presence of fat, liver tissue and iodine and the “Virtual Unenhanced” on water, air and iodine, as designed for less fatty tissue. The algorithm determines the content of each material and for each CT voxel [7]. Each voxel`s attenuation at different energies is fit to these basis materials and therefor an image without iodine can be virtually created [16]. Therefore, four series for each patient were created: two VNC reconstructed from arterial phase with the liver-specific “LiverVNC” application (VNC_Liver_^art^) and the conventional “Virtual Unenhanced” (VNC_conv_^art^) application and two reconstructed from portal venous phase (VNC_Liver_^pv^, VNC_conv_^pv^). TNC-series had been previously created for clinical use as 70 keV virtual mono-energetic images (Fig. 2).

### Image analysis

Circular ROI were placed in the left and right liver lobe and spleen, each measuring about 5 cm². If necessary, slight adaptions in size had to be made to meet the margins of the organs. The ROIs were placed by author A-K.G., 4 years of experience in abdominal imaging, in the portal venous images, firstly, avoiding large vessels and focal lesions and manually transferred to the arterial and true non-contrast images on the same slice position. Minimal manual adjustments of slice selection were made to compensate for breathing, if necessary. ROI placement was supervised by J.A.D, years of experience in abdominal imaging, and adjustments were made, if necessary. The mean attenuation values and standard deviation (SD) for VNC_conv_, VNC_Liver_ for arterial and portal venous phase and for TNC were obtained. The attenuation values of the right and left lobe on each image were averaged for each patient.

### Statistical analysis

Statistical analyses were performed using SPSS Statistics 28 (IBM, Armonk, New York, USA), MedCalc 2023 Software (MedCalc Software Ltd, Ostend, Belgium) and Excel 2016 (Microsoft, Redmond, Washington, USA). If not stated otherwise, all data were presented as mean ± SD of the mean or with 95% confidence interval (CI) as individually indicated. Graphical approach was used to assess normal distribution.

Offsets between attenuation values for TNC and VNC_Conv_ or VNC_Liver_ images were assessed by two-sided paired t-test. If normal distribution was not met, Wilcoxon signed-rank test was applied. Bland Altman plots were used for visualization. P-values ≤ 0.05 were considered to indicate statistically significant differences.

The impact of the BMI on the attenuation parameters was subsequently investigated through a median split. In order to evaluate the discrepancy between the two groups, the Student’s t-test and the Mann-Whitney U test were employed as appropriate statistical tools. Stratified for the application tool (TNC, VNC_conv_, VNC_Liver_) and for arterial and portal venous phase the differences in attenuation values were assessed graphically using the Passing Bablok regression, a non-parametric technique for comparing diagnostic results. Passing Bablok regression is not sensitive to outliers and does not require measurements errors to be normally distributed [17, 18]. In addition to this, the Passing Bablok regression has been shown to produce results that are comparable to those of the Deming Regression [19]. The confidence intervals of the slope and intercept for the function of TNC- VNC_conv_ or VNC_Liver_ were derived from arterial as well as portal venous phase were calculated. Should the CI of the slope contain zero and the CI of the intercept contain 1, no significant difference between the two methods can be concluded [17].

## Results

### Patients, scan parameters and radiation dosage

Retrospectively, 46 consecutive patients who underwent multiphasic photon counting CT scans of the abdomen between April 2021 and February 2023 were primarily included. After evaluating the specific exams, 42 patients met the inclusion criteria. The exclusion of one patient was due to the presence of advanced metastatic liver disease, with no remaining healthy liver tissue. Two patients were excluded due to a history of splenectomy, and one patient was excluded due to improper timing of phase contrast imaging (Fig. 1).

The mean age of the study population was 70.02 years ± 10.21 years. The majority of patients were male (*n* = 33). Only 9 patients were female. The mean BMI was 25.93 kg/m² ± 5.63 kg/m². DLP and CTDI_vol_ reflect truncal and abdominal scan ranges. Patient characteristics and radiation doses of the different scan protocols are presented in Table 1.

**Table 1:** Patient characteristics and radiation dose

Parameters	Value ± SD		
Mean age, years	70.02 ± 10.21		
Mean BMI, kg/m^2	25.93 ± 5.63		
No. patients ≥ 30 kg/m^2	7		
**Phase**	**True non-contrast**	**arterial**	**portal venous**
Mean DLP average, mGy*cm	277.45 ± 208.87	194.43 ± 105.86	284.34 ± 231.99
Mean DLP abdominal, mGy*cm	298.27 ± 212.61	208.37 ± 116.27	341.29 ± 243.94
Mean DLP truncal, mGy*cm	218.76 ± 195.27	155.17 ± 55.63	123.86 ± 61.23
Mean CTDI_vol_ average, mGy	8.56 ± 5.02	6.08 ± 3.82	6.71 ± 3.95
Mean CTDI_vol_ abdominal, mGy	9.90 ± 4.67	7.39 ± 3.61	8.10 ± 3.53
Mean CTDI_vol_ truncal, mGy	4.79 ± 4.08	2.39 ± 0.63	2.79 ± 1.91

### TNC vs. VNC

CT-values of liver parenchyma and spleen for TNC, VNC_conv_^art^, VNC_conv_^pv^, VNC_Liver_^art^, VNC_Liver_^pv^ are provided in Table 2.

**Table 2:** Mean values of the liver parenchyma

Mean attenuation (HU) ± SD	liver	spleen
TNC	59.01 ± 7.89	52.57 ± 3.34
VNC_conv_^art^	58.40 ± 8.11	47.90 ± 7.63
VNC_conv_^pv^	57.77 ± 8.84	48.96 ± 8.57
VNC_Liver_^art^	59.96 ± 9.01	48.15 ± 8.22
VNC_Liver_^pv^	59.36 ± 9.71	49.86 ± 9.19

**Fig. 3:**
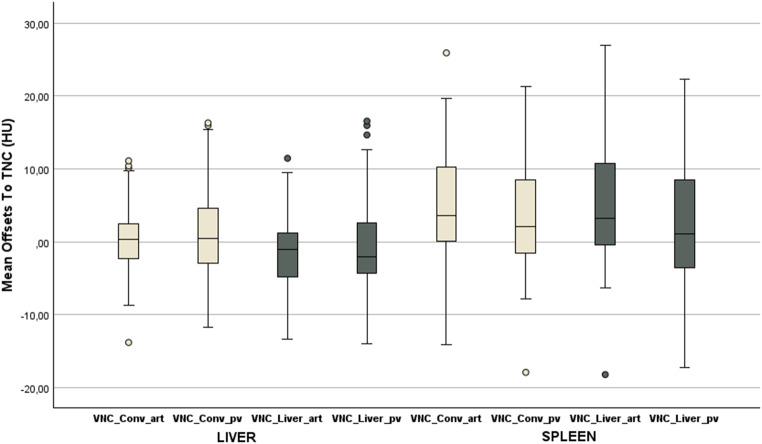
Boxplot of mean offsets to TNC of liver and spleen parenchyma. Note: The mean offsets to TNC for each organ and each true non-contrast image is displayed

It was observed that there were only minor differences in the mean attenuation of the liver and spleen parenchyma across the various contrast phases and post-processing applications. Mean offsets are presented in Table 3. In general, the mean offsets for the liver attenuation were not exceeding 5 HU in difference. VNC images from the conventional VNC algorithm generally showed a positive difference and images from the liver-specific algorithm a negative difference. The average differences in attenuation of the spleen were less than 5 HU and exclusively positive. A total of 5 patients accounted for differences > 10 HU in VNC_conv_^art^-TNC and VNC_Conv_^pv^-TNC comparisons. For VNC_Liver_^art/pv^, a total of 4 different patients accounted for deviations > 15 HU. No significant difference of the attenuation of liver parenchyma could be seen between TNC and each VNC (all *p* ≥ 0.05) as shown in Table 3 (see also Fig. 3). However, a significant difference for spleen attenuation was seen for every VNC despite VNC_Liver_^pv^ (Table 3; Fig. 3). A patient with a clinically significant deviation had a BMI of 37 kg/m².

**Table 3:** Absolute differences of Attenuation of liver and spleen parenchyma

	Liver Attenuation±SD (HU)	Two-sided *p*-value (Student’s T/ Wilcoxon)	Spleen Attenuation±SD (HU)	Two-sided *p*-value (Student’s T/ Wilcoxon)
TNC- VNC_conv_^art^				
Mean offset	0.62±5.23	0.45/0.69	4.67±7.97	<0.001/<0.001
≥10 HU	10.53±0.51 (3 patients, 7.1%)		15.07±4.70 (11 patients, 26.2%)	
≥15 HU	0 (0 patients)		18.94±4.35 (5 patients, 11.9%)	
TNC- VNC_conv_^art^				
Mean offset	1.24±6.67	0.24/0.45	3.62±8.38	0.008/0.01
≥10 HU	14.88±1.42 (5 patients, 11.9%)		16.74±4.48 (8 patients, 19%)	
≥15 HU	15.88±0.45 (3 patients, 7.1%)		19.68±2.09 (5 patients, 11.9%)	
TNC- VNC_Liver_^art^				
Mean offset	-0.94±5.59	0.28/0.16	4.42±8.43	0.002/0.002
≥10 HU	11.45 ±0 (1 patient, 2.4%)		15.35±4.99 (11 patients, 26.2%)	
≥15 HU	0 (0 patients)		20.53±4.73 (4 patients, 9.5%)	
TNC- VNC_Liver_^art^				
Mean offset	-0.35±6.99	0.75/0.29	2.71±9.03	0.06/0.12
≥10 HU	14.08±2.45 (5 patients, 11.9%)		19.45±2.95 (6 patients, 14.3%)	
≥15 HU	16.25±0.42 (2 patients, 4.8%)		19.45±2.95 (6 patients, 14.3%)	

**Fig. 4:**
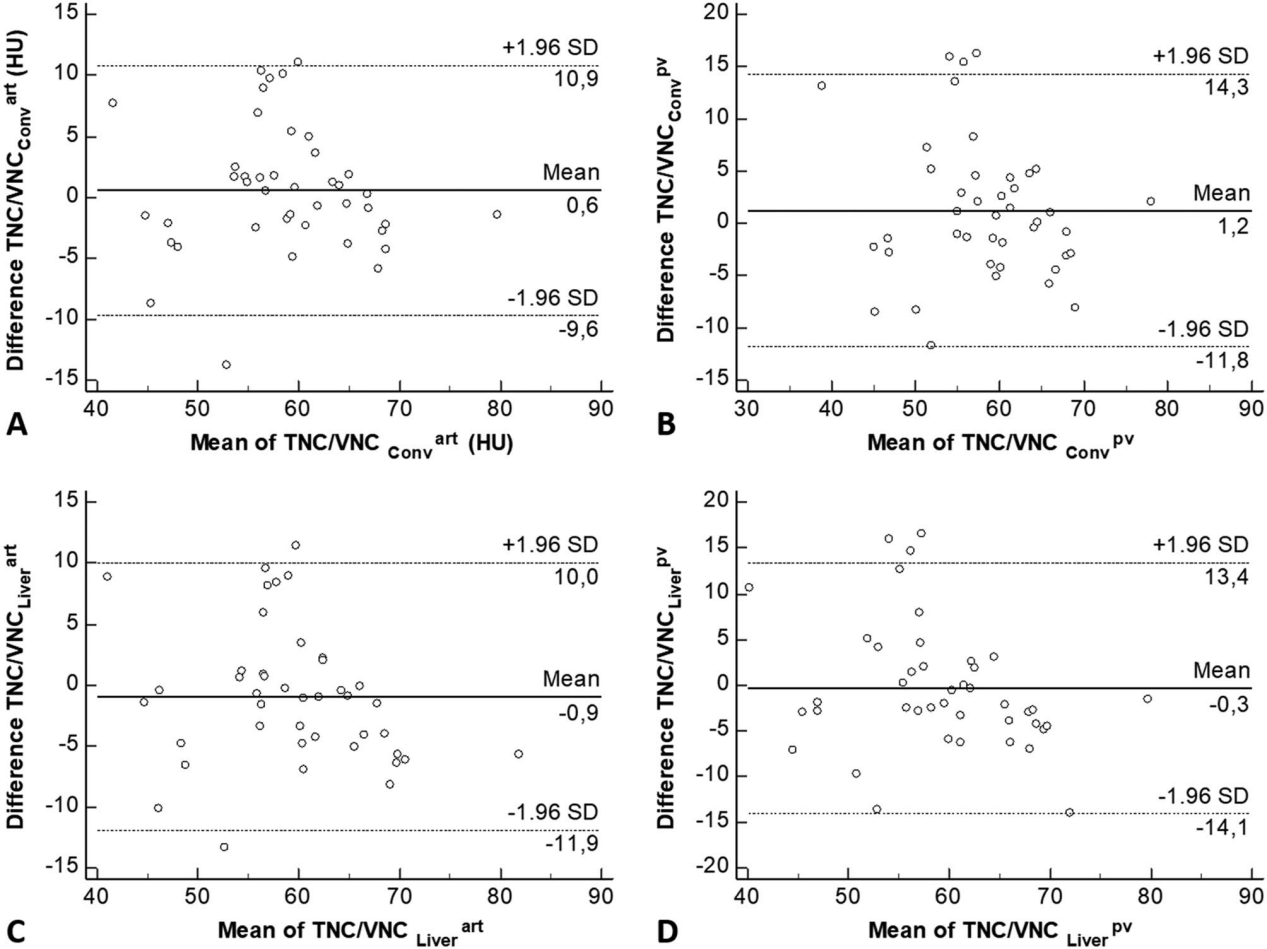
Bland-Altman Plots. Bland-Altman Plots showing the relationship between the differences of measurements on TNC and VNC_conv_^art/pv^ (**A**; **B**) or VNC_Liver_^art/pv^ (**C**; **D**) for the liver parenchyma. Measurements spread around the mean of the differences and most measurements lie between ± 1.96 standard deviations of the difference

Bland-Altman Plots showed no systematic difference between TNC and each VNC and scan phase (Fig. 4).

Pearson`s correlation coefficient *r* was 0.79 for TNC-VNC_conv_^art^, 0.69 for TNC-VNC_conv_^pv^, 0.79 for TNC-VNC_Liver_^art^ and 0.70 for TNC-VNC_Liver_^pv^ (all *p* < 0.001) with significant and good correlation.

### BMI

The BMI of 25 patients was available and median split was performed (median BMI of 25.71 kg/m^2^). 7 patients’ BMI was above 30 kg/m^2^ and the highest BMI was 37 kg/m^2^. Mean liver attenuation of group 1 (BMI lower than median) and 2 (higher than median) was 60.71 ± 9.19/ 58.73 ± 7.22 HU (TNC), 60.00 ± 9.18/ 58.37 ± 8.41 HU (VNC_conv_^art^), 59.28 ± 9.42/ 58.19 ± 9.01 HU (VNC_conv_^pv^), 61.53 ± 10.45/ 59.87 ± 9.41 HU (VNC_Liver_^art^) and 60.83 ± 10.40/ 60.27 ± 9.39 HU (VNC_Liver_^pv^).

**Fig. 5:**
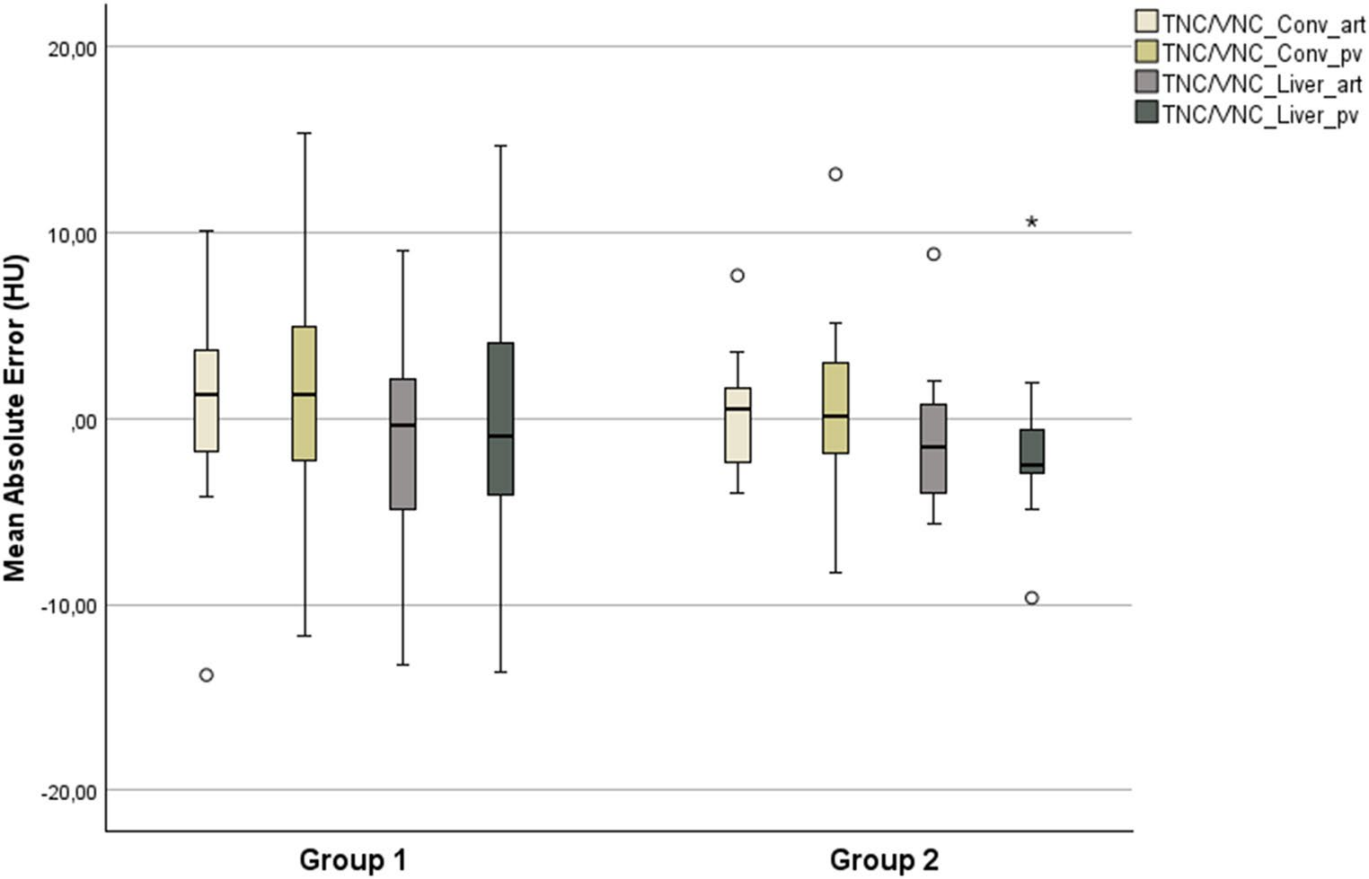
Mean absolute errors to TNC of the liver parenchyma for group 1 and 2 are displayed. Offsets between TNC and VNC from different scan phases and post-processing algorithms are shown. * shows an extreme value

In the first group mean offsets between TNC and VNC_conv_^art^, VNC_conv_^pv^, VNC_Liver_^art^ or VNC_Liver_^pv^ were 0.70 HU ± 6.26 HU; 1.43 HU ± 6.78 HU; -0.82 HU ± 6.19 HU and − 0.13 HU ± 7.30 HU. In the second group, offsets for the attenuation values were 0.36 HU ± 3.09 HU (VNC_Conv_^art^), 0.55 HU ± 5.13 HU (VNC_conv_^pv^), -1.14 HU ± 3.82 HU (VNC_Liver_^art^) and − 1.54 HU ± 4.62 HU (VNC_Liver_^pv^), with no significant difference between group 1 and 2 (p-values: VNC_conv_^art^ 0.51, VNC_conv_^pv^ 0.61, VNC_Liver_^art^ 0.68, VNC_Liver_^pv^ 0.45) (Table 4; Fig. 5).

**Table 4:** Mean offsets between TNC-VNC stratified for the BMI group

	BMI < 25.71 kg/m^2^	BMI ≥ 25.71 kg/m^2^	*p*-value (t-Test/ Mann-Whitney-Test)
Mean TNC-VNC_conv_^art^	0.70 ± 6.26	0.36 ± 3.09	0.86/ 0.53
Mean TNC-VNC_conv_^pv^	1.43 ± 6.78	0.55 ± 5.13	0.71/ 0.61
Mean TNC-VNC_Liver_^art^	-0.82 ± 6.19	-1.14 ± 3.82	0.87/ 0.69
Mean TNC-VNC_Liver_^pv^	-0.13 ± 7.30	-1.54 ± 4.62 HU	0.57/ 0.47

**Fig. 6:**
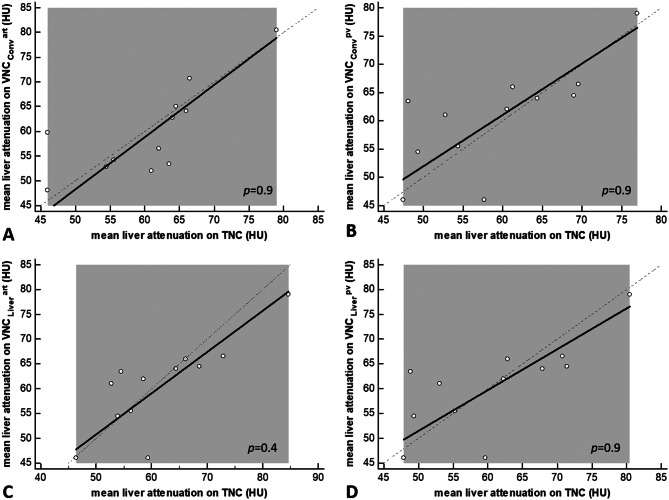
Passing-Bablok Regression model for group 1. Note: Passing-Bablok Regression model for (**A**) VNC_conv_^art^, (**B**) VNC_conv_^pv^, (**C**) VNC_Liver_^art^, (**D**) VNC_Liver_^pv^. According to the confidence intervals reported, the diagram shows no systematic or proportional difference

**Fig. 7:**
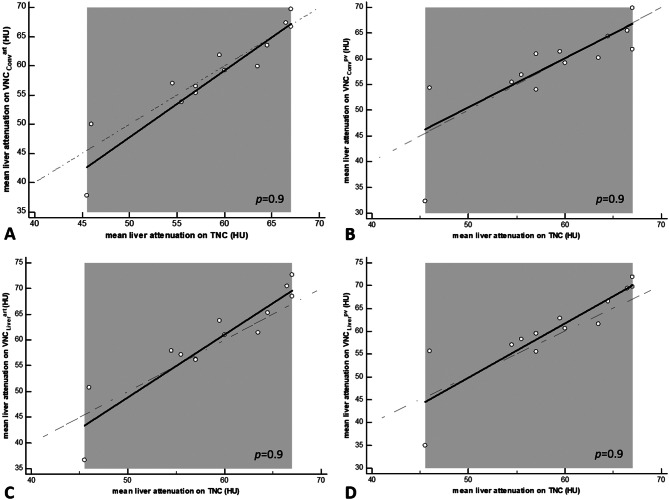
Passing-Bablok Regression model for group 2. Note: Passing-Bablok Regression model for (**A**) VNC_conv_^art^, (**B**) VNC_conv_^pv^, (**C**) VNC_Liver_^art^, (**D**) VNC_Liver_^pv^. According to the confidence intervals reported, the diagram shows no systematic or proportional difference

As demonstrated in Figs. 6 and 7, the scatter diagrams for the Passing Bablok regression indicate visually good agreement within each BMI-group. Furthermore, no significant difference was demonstrated between TNC and VNC_conv_^art^, VNC_conv_^pv^, VNC_Liver_^art^ or VNC_Liver_^pv^ retrospectively within both groups (Supplemental Table 1).

## Discussion

The aim of this study was to evaluate PCD-CT-VNC images using conventional and liver-specific reconstruction algorithms across arterial and portal venous phases. Furthermore, the impact of BMI on attenuation values was examined.

The topic of VNC has been evaluated many times in the literature with regard to DECT and PCD-CT [7, 9, 11], but essential aspects such as the exact algorithm used are not often mentioned. When results regarding the accuracy of VNC images sometimes deviate, it is necessary to investigate whether these discrepancies can be explained by this. Furthermore, it is known that excess weight influences attenuation values in CT. However, it has not yet been investigated whether this is an influencing factor for the deviation of VNC images in either DECT or PCD-CT. We investigated mean offsets between VNC and TNC. *Mergen et al.* demonstrated offsets of 1.5/1.4 HU on arterial VNC for right and left liver lobe and 1.6/1.5 HU on portal venous VNC [11]. In our study, mean offsets were 0.62/-0.94 HU (VNC_conv_^art^/VNC_Liver_^art^) and 1.24/-0.35 HU (VNC_conv_^pv/VNC^_Liver_^pv^). In contrast to the findings of our study, in which 7.14/2.38% (VNC_conv_^art^/VNC_Liver_^art^) and 11.90/11.90% (VNC_conv_^pv^ /VNC_Liver_^pv^) of patients demonstrated an offset greater than 10 HU offset, *Mergen et al.* detected no patients with a difference greater than 10 HU [11]. Moreover, in the present study, no patient demonstrated an offset greater than 15 HU on the arterial VNCs. However, 3/2 of the 42 patients examined showed this on VNC_conv_^pv^/VNC_Liver_^pv^. A total of five patients exhibited differences greater than 10 HU in the comparisons between VNC_conv_^art^-TNC and VNC_conv_^pv^-TNC. For VNC_Liver_^art/pv^, deviations exceeding 15 HU were observed in four distinct patients. One of these patients had metastatic disease but metastases were small and distant from the measurements. The others did not have any noteworthy conditions. Consequently, no consistent pathological factors could be identified to account for the higher HU differences. These variations are therefore interpreted as expected fluctuations inherent to the VNC algorithms, as previously reported and discussed within this manuscript. Overall, no statistically significant difference between VNC_conv_^pv^ / VNC_Liver_^pv^ and TNC were detected in our study. A plausible explanation may be the difference in sample size, as *Mergen et al.* evaluated 100 patients compared to 42 in our study. The larger cohort in their analysis may place our findings into perspective. Moreover, *Mergen et al.* did not specify the reconstruction algorithm employed, which may further account for differences. Notably, differences in splenic attenuation between TNC and VNC in both the arterial and portal venous phases were comparable to those reported by *Mergen et al.*, remaining below 5 HU and without clinical relevance [11].

*Schoenbeck et al.* investigated the offsets of PCD-CT TNC and portal venous VNC. Comparable to our study 87/89% (right and left liver lobe) of patients showed less than 10 HU and 98/ 96% less than 15 HU offset. Mean offsets of the spleen were consistent with our findings of 3.62/2.71 HU (VNC_conv_^pv^/VNC_Liver_^pv^).

In contrast to our findings, *Niehoff et al.* reported greater differences in liver parenchyma attenuation on VNC images derived from PCD-CT. They observed a mean offset of 8.90 HU in the arterial VNC phase and 10.96 HU in the portal venous VNC phase, both showing statistically significant differences compared to TNC [9]. In more than 50% of their cases, the attenuation offset exceeded 10 HU; however, in approximately 70% of cases, the difference remained below 15 HU. These results underscore the need for caution when interpreting VNC images. Although both studies employed a ROI of 5 cm² within the liver, *Niehoff et al.* did not specify the exact placement of the ROI—whether measurements were obtained from both lobes, and whether large vessels or focal lesions were excluded—which could influence attenuation values. Like in our study, a significant difference could be seen for the spleen. An explanation could be that the post-processing algorithms are adapted for the liver parenchyma and therefore miscalculated the iodine content of the vascularized spleen.

In literature, the exact reconstruction algorithm is often not mentioned. *Schoenbeck et al.* investigated conventional and liver-specific algorithm and detected significant but not clinically relevant difference for the liver and spleen for both algorithms on portal venous phase VNC. Slightly better results showed the liver specific algorithm [14]. In our study images reconstructed with “LiverVNC” algorithm showed a slight negative offset whereas “Virtual Unenhanced” images a slight positive offset but within 10 HU of deviation to TNC. In concordance to *Schoenbeck et al.* both reconstruction algorithms demonstrated no clinically relevant offset to TNC [14].

Furthermore, BMI is known to influence the quality of CT images and optimized scanning protocols are required for obese patients in order to reduce radiation dose while maintaining diagnostic image quality [20]. Consequently, we assessed whether BMI impacts attenuation values of VNC images. The median BMI in our cohort (*n* = 25) was 25.71 kg/m², with a maximum BMI of 37 kg/m². This median value lies at the upper limit of the normal weight range and effectively separates the cohort into normal/underweight versus overweight/obese subgroups. Seven patients presented with a BMI ≥ 30 kg/m². This distribution allows, to some extent, a representative comparison between patients with normal weight and those who are overweight or obese. No statistically significant differences were observed in the mean attenuation offsets between TNC and VNCs comparing patients above and below the median BMI. Within each BMI group we demonstrated good agreement between the TNC and all VNC algorithms and contrast phases. These findings suggest that BMI may not have a clinically relevant effect on the performance of VNC post-processing algorithms, although it is important to note that our study did include only 7 individuals with severe obesity. Therefore, these findings should be treated with caution, and subsequent studies involving a larger proportion of patients with a higher BMI are necessary to confirm these results and enable them to be generalized. *Durieux et al.*, using the same post-processing algorithms as in our study, demonstrated for all regions and for both post-processing algorithms except for the liver, spleen and paraspinal muscle on portal venous VNCs significantly differences. Moreover, these differences were significantly and positively correlated to the BMI, except for the liver on arterial conventional VNC with a non-significant correlation [7]. Their findings for the liver are consistent with our results and support the conclusion that liver attenuation in VNC images is not significantly influenced by BMI. The significant correlation observed by *Durieux et al.* in arterial “Liver VNC” images—unlike in our cohort—may be attributable to the improved spectral resolution of PCD-CT, as DECT is known to be affected by spectral overlap [21]. Additionally, *Hagen et al.* investigated images quality and dose reduction of PCD-CT and DECT in obese patients. They demonstrated significantly better contrast-to-noise ratio on PCD-CT images with significantly reduced radiation dose for parenchymatous organs and vessels [22]. The findings of this study, when viewed in conjunction with literature on the subject, suggest that the VNC images demonstrate satisfactory diagnostic capabilities, with caution for obese patients.

There are certain limitations to this study. This study is a single centre study with one PCD-CT scanner available and no opportunity to compare the results to other scanners to put results into context. The retrospective setting of this study causes a certain degree of selection bias. In an attempt to reduce bias, the authors made the decision to include all CT scans that were available within the stated time frame. This decision was made with the intention of creating a heterogeneous collective of patients. As mentioned above patients with diffuse metastatic disease were excluded due to no apparent liver tissue. Placing the ROIs, focal lesions were avoided and were not further investigated as the aim of the study was to investigate the general performance of VNCs. As a shortcoming of this study, no subgroup analysis regarding possible underlying liver disease was conducted. As this was not decidedly addressed in this study, this could possibly influence our findings and further investigations are needed. Furthermore, only 7 obese patients were included due to the retrospective character of the study design and therefor our results do not fully reflect the influence of BMI on VNCs. Our findings should be confirmed by prospective studies with greater sample size and a dedicated analysis in further studies for underlying liver disease like cirrhotic vs. non-cirrhotic.

In conclusion, VNC images reconstructed from the first commercially available PCD-CT system represent a valid alternative to TNC images. Notwithstanding the fact that VNC images reconstructed from arterial and portal venous phases exhibited, on average, no clinically significant difference compared to TNC images, it is nevertheless recommended that caution be exercised when applying VNC images. Outliers of differences ≥ 15 HU were reported and have the potential to significantly impact image interpretation. Nevertheless, these results are encouraging and suggest that PCD-CT VNC images may be a viable alternative to TNC images in clinical practice. It is evident that the omission of TNC images in CT protocols would result in a substantial reduction in patient radiation exposure. Furthermore, this approach would result in a reduction of time for personnel involved in clinical routines, primarily due to the elimination of the initial unenhanced phase and, secondly, the prevention of repeated scans for the retrospective generation of TNC.

## Conclusion

It is evident that PCD-CT-derived VNC images generally constitute a corresponding alternative to TNC images. However, caution is advised in the interpretation of images, as there are outliers with differences exceeding 15 HU are present. In general, the mean values obtained from the analysis of, VNC images reconstructed from arterial and portal venous phases employing both the liver-specific and general VNC reconstruction algorithm did not demonstrate any clinically significant difference when compared with TNC images. Furthermore, no significant discrepancy was observed in the utilisation of the conventional and the liver-specific algorithm. The findings of this study demonstrated that, within the limitations of the study, the patients’ BMI did not have a significant impact on the VNC images.

## Supplementary Information

Below is the link to the electronic supplementary material.

Supplementary Material 1

## Data Availability

The data analyzed and presented in this study are available from the corresponding author upon reasonable request.

## Abbreviations

BMI: Body Mass Index
CI: Confidence Intervall
DECT: Dual Energy CT
DLP: Dose Length Product
CTDIVol: Volumetric CT Dose Index
HU: Hounsfield Units
PCD-CT: Photon Counting Detector Computed Tomography
QIR: Quantum iterative reconstruction
ROIs: Regions of interest
SD: Standard deviation
TNC: True non-contrast
VNC: Virtual non-contrast
VNC_Conv_^art^: VNC reconstructed from arterial phase and conventional algorithm
VNC_Conv_^pv^: VNC reconstructed from portal venous phase and conventional algorithm
VNC_Liver_^art^: VNC reconstructed from arterial phase and liver specific algorithm
VNC_Liver_^pv^: VNC reconstructed from portal venous phase and liver specific algorithm
