# Supplementary material for: Photon counting detector CT-derived virtual non-contrast images of the liver: comparison of conventional and liver-specific algorithms across arterial and portal venous phase scans
Source: BMC Med Imaging. 2025 Aug 4;25:311. doi: 10.1186/s12880-025-01849-0 (PMC12323134; doi:10.1186/s12880-025-01849-0)
Supplement: Supplementary file 1 — Supplementary Material 1 [file 12880_2025_1849_MOESM1_ESM.pdf]

Supplement

Supplemental Table 1. BMI evaluation

**Group 1**	**VNC_{Conv}^{art}**	**VNC_{Conv}^{pv}**	**VNC_{Liver}^{art}**	**VNC_{Liver}^{pv}**
95% CI intercept	-57.4 – 26.6	-63.5 – 23.6	-88.0 – 15.1	-111.3 – 21.8
95% CI slope	0.5 – 1.9	0.6 – 2.0	0.8 – 2.4	0.6 – 2.8
Group 2				
95% CI intercept	-30.8 – 9.1	-44.8 – 27.9	-38.8 – 10.8	-37.4 – 13.9
95% CI slope	0.8 – 1.5	0.5 – 1.7	0.8 – 1.7	0.8 – 1.6

Note. Passing-Bablok-Regression model showed no constant or proportional difference. If the 95%-CI of the intercept does include 0 and the 95%-CI of the slope include 1, no significant difference could be concluded.
